# Study on the short-term effects of increased alcohol and cigarette consumption in healthy young men’s seminal quality

**DOI:** 10.1038/srep45457

**Published:** 2017-04-03

**Authors:** Joana Vieira Silva, Daniel Cruz, Mariana Gomes, Bárbara Regadas Correia, Maria João Freitas, Luís Sousa, Vladimiro Silva, Margarida Fardilha

**Affiliations:** 1 Laboratory of Signal Transduction, Department of Medical Sciences, Institute of Biomedicine – iBiMED, University of Aveiro, Campus Universitário de Santiago, 3810-193, Aveiro, Portugal; 2 Ferticentro – Center for Fertility Studies, Praceta Prof. Robalo Cordeiro (Idealmed), Circular Externa de Coimbra, 3020-479 Coimbra, Portugal

## Abstract

Many studies have reported a negative impact of lifestyle factors on testicular function, spermatozoa parameters and pituitary-gonadal axis. However, conclusions are difficult to draw, since studies in the general population are rare. In this study we intended to address the early and late short-term impact of acute lifestyle alterations on young men’s reproductive function. Thirty-six healthy male students, who attended the Portuguese academic festivities, provided semen samples and answered questionnaires at three time-points. The consumption of alcohol and cigarette increased more than 8 and 2 times, respectively, during the academic festivities and resulted in deleterious effects on semen quality: one week after the festivities, a decrease on semen volume, spermatozoa motility and normal morphology was observed, in parallel with an increase on immotile spermatozoa, head and midpiece defects and spermatozoa oxidative stress. Additionally, three months after the academic festivities, besides the detrimental effect on volume, motility and morphology, a negative impact on spermatozoa concentration was observed, along with a decrease on epididymal, seminal vesicles and prostate function. This study contributed to understanding the pathophysiology underlying semen quality degradation induced by acute lifestyle alterations, suggesting that high alcohol and cigarette consumption are associated with decreased semen quality in healthy young men.

Infertility affects about 15% of couples attempting to conceive and in half of these cases the cause is related to male reproductive issues^1^. A large percentage of male infertility cases are idiopathic (~40%), though in the recent years the influence of oxidative stress (OS) in decreased semen quality has been discussed^2^,^3^. The excessive production of reactive oxygen species (ROS) in the male reproductive system raises concern due to their potential toxic effects on sperm quality and function, which may ultimately lead to male infertility^3^,^4^. Several environmental and lifestyle factors, such as alcohol and cigarette consumption, are known to increase the levels of ROS in semen^4^,^5^,^6^,^7^. Many studies have reported a negative impact of these lifestyle factors on testicular function, spermatozoa parameters and pituitary-gonadal axis dysfunction^6^,^8^,^9^,^10^,^11^,^12^,^13^. However, conclusions are difficult to draw, since several studies have shown contradictory results. Studies conducted on the general population are rare, mainly due to difficulties in sample collection and patient follow-up. Most studies are conducted in infertile men or individuals that attend medical care, which introduces bias; some included only infertile patients, while others included both infertile and fertile men, precluding any meaningful comparisons between the different data sets.

In Portugal, alcohol consumption among individuals with more than 15 years of age has been increasing (12.9 liters on average/individual/year)^14^, whilst around 32.4% of male Portuguese older than 15 years smoked^14^. There is a strong tradition of acute abuse of alcohol, cigarette and drug during the Portuguese academic festivities. This offers exceptional conditions for studying this problem, since a large number of young male individuals voluntarily expose themselves to high quantities of harmful factors during a very well defined and limited period of time, having had a relatively healthy lifestyle before and after the exposure.

In this study we intended to assess the early and late short-term impact of acute lifestyle alterations, namely alcohol and cigarette consumption, in human sperm basic parameters and oxidative balance. Furthermore, given the importance of the seminal plasma for spermatozoa maturation, nutrition and protection, the function of epididymal and accessory glands was also evaluated.

## Methods

### Study design and participants

**Figure 1:**
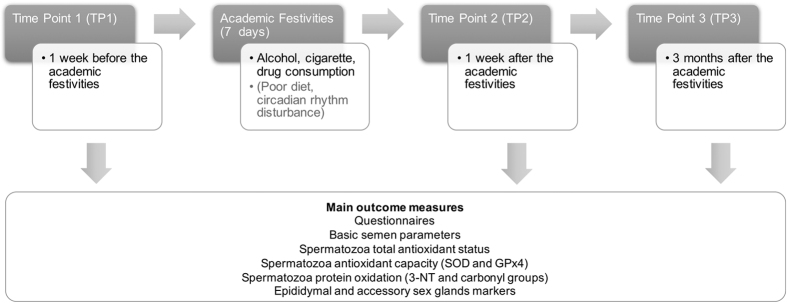
Study design and main outcome measures. Unmeasured confounders occurring during the academic festivities include factors such as diet and circadian rhythm alterations. TP, time-points.

The effects of several lifestyle factors on semen quality were studied in samples from healthy young male volunteers, in reproductive age, during three well defined moments: before (time point 1 - TP1), one week (time point 2 - TP2) and three months (time point 3 - TP3) after the Portuguese academic festivities (a 7 days event), where there is a strong tradition of alcohol, cigarette and drug abuse (Fig. 1). Only volunteers who provided a sample in all three TPs were considered.

The study was advertised on the Campus of University of Aveiro and only students from that University were accepted as volunteers. The advertisement clearly stated the hypothesis and main goals of the study and no monetary compensation was applied. All participants received clear written instructions concerning the sample collection and answered an assisted fulfilment questionnaire each time a sample was provided. The questionnaires contained questions regarding abstinence length, past/recent illnesses, medication and alcohol, cigarette and drugs (such as cannabis and cocaine) consumption. The alcohol consumption parameter was defined as an estimated total amount of alcohol (in grams). In TP1 and TP3, this parameter considered the alcohol consumed, per week, during the month prior to the academic festivities. TP2 reflected the alcohol consumed during the academic festivities week. The cigarette consumption parameter was defined as an estimated total amount of nicotine (in milligrams). In TP1 and TP3, this parameter considered the nicotine consumed, per week, during the month prior to the academic festivities. TP2 reflected the nicotine consumed during the academic festivities week. All data was anonymized.

### Ethical approval

This study was approved by the Ethics and Internal Review Board of the Hospital Infante D. Pedro E.P.E., Aveiro, Portugal (Process number: 36/AO) and was conducted in accordance with the ethical standards of the Helsinki Declaration. All participants received clear written instructions concerning the study design and signed informed consent allowing the samples to be used for scientific purposes.

### Semen sample collection, basic semen analysis and sperm preparation

Semen samples were obtained by masturbation into a sterile container and delivered for basic semen analysis within 30 minutes. All samples were analyzed according to the World Health Organization criteria by experienced technicians^15^. Briefly, after complete liquefaction of the semen samples at 37 °C, during approximately 30 minutes, a macroscopic examination was performed. The microscopic examination included the analysis of spermatozoa motility, concentration and morphology. All microscopy analysis was performed using a Zeiss Primo Star microscope (Carl Zeiss AG, Germany). Semen samples were washed 3 times in PBS. In the first wash, the seminal plasma was recovered and used for further tests. Sperm cells extracts were ressuspended in 1% SDS. Protein concentration was measured using a bicinchoninic acid assay (Pierce BCA Protein Assay Kit, Thermo Fisher Scientific, USA), and final absorbance was measured at 562 nm in a microplate reader (Infinite^®^ 200 PRO series, Tecan Trading AG, Switzerland).

### Western blot analysis of antioxidant enzymes superoxide dismutase 1 (SOD1) and glutathione peroxidase 4 (GPx4) expression

Spermatozoa extracts were sonicated, boiled and equal amounts of protein (50 μg) were loaded in a 10% SDS-PAGE gel, and transferred to a nitrocellulose membrane. Immunodetection was performed using mouse anti-SOD1, 1:1000 (MABC864, Merck Milipore, Darmstadt, Germany) and rabbit anti-GPx4 (ABC269, Merck Milipore, Darmstadt, Germany), followed by incubation with the corresponding infrared secondary antibody (1:5000, LI-COR Biosciences UK Ltd, Cambridge, UK). Beta-tubulin (Merck Milipore, Darmstadt, Germany) was used as a loading control. The membrane was scanned using an Odyssey Infrared Imaging System (LI-COR Biosciences, Cambridge, UK).

### Carbonyl group (CG) determination by Slot blot

Spermatozoa extracts containing 30 μg of protein were derivatized with 20 mM of 2.4-dinitrophenylhydrazine dissolved in 10% trifluoroacetic acid and neutralized with 1.5 volumes of 2 M of Tris with 18% of mercaptoethanol. To perform the slot blot, the sample was diluted to 0.002 μg/μL and applied into a nitrocellulose membrane inside the slot blot device (BioRad Portugal, Sintra, Portugal). The membrane was incubated with rabbit anti-DNP, 1:5000 (MAB2223, Merck KGaA, Darmstadt, Germany) followed by anti-rabbit infrared secondary antibody (1:5000, LI-COR Biosciences UK Ltd, Cambridge, UK). The detection was performed in the Odyssey Infrared Imaging System (LI-COR Biosciences, Cambridge, UK).

### 3-Nitrotyrosine (3-NT) determination in spermatozoa by Slot blot

Spermatozoa extracts were diluted in 1xTBS to a final concentration of 1 ηg/μL. The samples were applied in the slot blot (BioRad Portugal, Sintra, Portugal) into a nitrocellulose membrane. Next, the membrane was incubated with rabbit anti-nitrotyrosine, 1:1000 (DAM1514077, Merck KGaA, Darmstadt, Germany) followed by anti-rabbit infrared secondary antibody (1:5000, LI-COR Biosciences UK Ltd, Cambridge, UK). The detection was performed in the Odyssey Infrared Imaging System (LI-COR Biosciences UK Ltd, Cambridge, UK).

### Total antioxidant status (TAS)

The concentration of antioxidants in sperm cells was measured using the Total Antioxidant Status (TAS) assay (NX2332, Randox laboratories, Crumlin, Nothern Irland). The protocol provided by the manufacturer was modified so that a microplate could be used (reagents and sample quantities were scaled down).

### Biochemical analysis of epididymal and accessory sex glands markers

Biochemical markers of the epididymal function (neutral α-glucosidase, NAG), prostatic function (citric acid), and seminal vesicle function (fructose) were assessed in the seminal plasma. Total NAG activity was measured using a commercially available kit EpiScreen Plus-α-glucosidase assay (FertiPro, Beernem, Belgium) according to the instructions given by the manufacturer. To quantify the amount of fructose in the seminal plasma the Fructose Test assay (FertiPro, Beernem, Belgium) was used according to manufactures’ instructions. The level of citric acid in seminal plasma was measured using the Citric Acid Test (FertiPro, Beernem, Belgium) according to manufactures instructions.

### Statistical analysis

A longitudinal study was conducted to analyze the impact of Portuguese academic festivities on human spermatozoa. A total of 36 volunteers, who attended the academic festivities, voluntarily participated in the study. The individuals were followed before (TP1) and after (TP2 and TP3) the academic festivities. The dataset size was considered reasonable since based on the central limit theorem it was possible to approximate the distribution of variables to a normal distribution. Initially an exploratory data analysis (EDA) was performed using graphical techniques (bar charts, box and scatter plots) and quantitative analysis (statistical measures, frequency and contingency tables) in order to characterize the sample, detect possible extreme outliers and measurement errors. To recognize the alterations for each parameter during follow-up periods, tests of the equality of means for paired samples: t-test (parametric test) and Wilcoxon Signed Rank test or Sign test (non-parametric tests) were performed. The assumptions of the paired difference tests were performed (Shapiro Wilks test and measures of skewness). The standardized mean effect size - Cohen’s d (difference between the means divided by the pooled SD) - was calculated. Cohen’s d is an appropriate effect size to accompany reporting of inferential testing and provides useful information for discussion (e.g. the size of effects reported in other studies). We defined effect sizes as “small, d = 0.2,” “medium, d = 0.5,” and “large, d = 0.8”. The significance level was set at 0.05 and the statistical analysis was conducted using the IBM SPSS Statistics Software 22.

## Results

**Figure 2:**
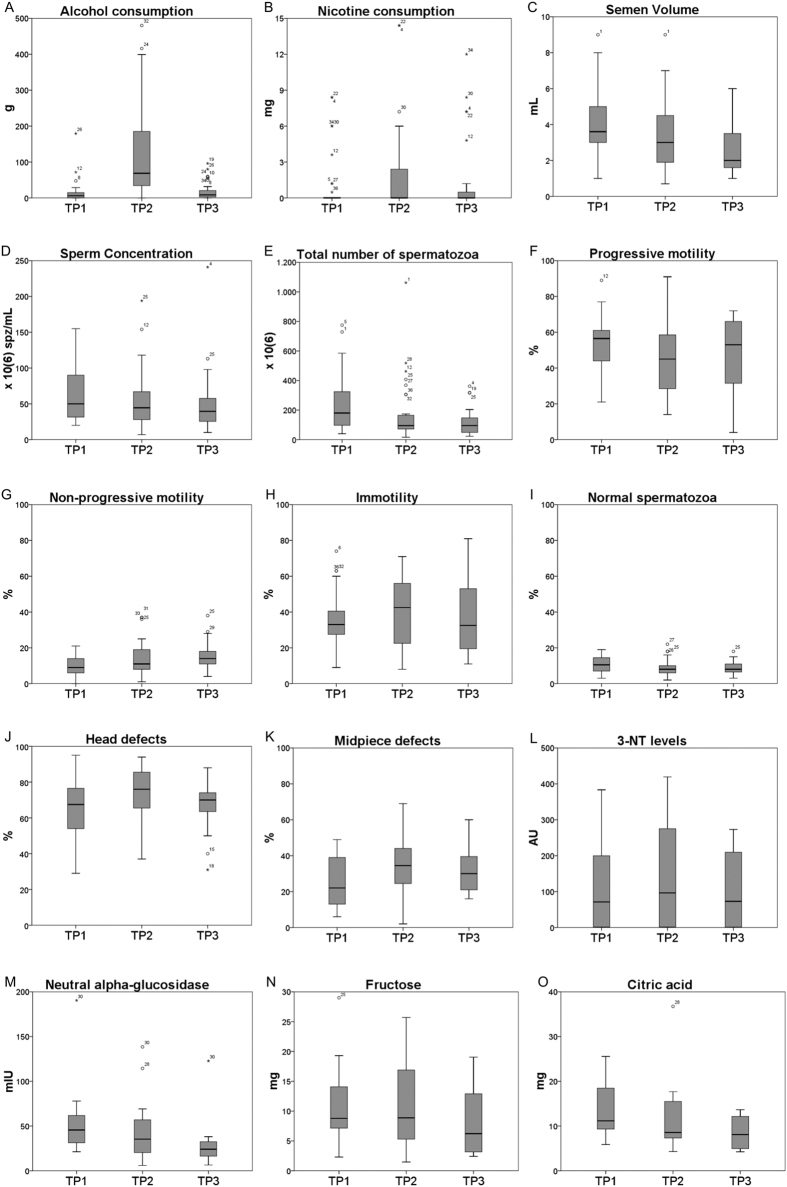
Box plot of the alcohol (**A**) and nicotine (**B**) consumptions, basic semen parameters (**C**–**K**), oxidative stress marker (**L**) and epididymal (**M**) and accessory sex glands (**N** and **O**) function markers at the different time points (TP). p-values of paired-samples tests are summarized in Table 2.

A total of 36 healthy Caucasian male students with the mean age of 22 ± 3 years were included in this study. No prior pregnancies, medical comorbidities, medications and drug use were reported among the participants included in the dataset. A descriptive analysis of the results is presented in Table 1. Only the statistically significant results are described below (Fig. 2, Table 2). All paired difference between the study time-points (TPs) are described in Supplementary Table S1.

### Impact of the academic festivities on the semen basic parameters and spermatozoa oxidative stress markers

The consumption of alcohol per day increased more than 8 times between TP1 and TP2. At TP3, the alcohol consumption values decreased to basal levels (TP1) (Fig. 2A; Table 2). At TP2, nicotine consumption increased significantly when compared with TP1 (2 times) (Fig. 2B; Table 2).

Differences in basic semen parameters were observed (Fig. 2C–K; Table 2). Semen volume presented a significant decrease over the TPs (18% from TP1 to TP2; 19% from TP2 to TP3; 33% from TP1 to TP3) (Fig. 2C, Table 2). Spermatozoa concentration and the total number of spermatozoa in the ejaculate decreased significantly from TP1 to TP3 (20% and 52%, respectively) (Fig. 2D and E; Table 2). The percentage of progressive motility decreased from TP1 to TP2 (15%) (Fig. 2F; Table 2). The percentage of non-progressive motile spermatozoa increased over the TPs (40% from TP1 to TP2; 50% from TP1 to TP3) (Fig. 2G; Table 2). The percentage of immotile spermatozoa significantly decreased from TP1 to TP2 (14%) (Fig. 2H; Table 2). Concerning spermatozoa morphology, the percentage of normal spermatozoa was significantly higher at TP1 when compared with both TP2 and TP3 (Fig. 2I; Table 2). Specifically, head and midpiece defects were higher in TP2 when compared with TP1 and TP3 (Fig. 2J and K; Table 2).

The levels of 3 nitrotyrosine (3NT) in spermatozoa was the only oxidative stress marker that showed a significant alteration (Fig. 2L; Table 2). In TP2, the levels of tyrosine residues which suffer nitration was 1.4 times higher than in TP3.

### Impact of the academic festivities on epididymal and accessory sex glands function

There was a significant decrease in the activity of neutral α-glucosidase (NAG) in the ejaculate from TP1 to TP3 (46%) as well as from TP2 to TP3 (35%) (Fig. 2M; Table 2). Concerning the total amount of fructose in the ejaculate, there was also a significant decrease from TP1 to TP3 (28%) as well as from TP2 to TP3 (23%) (Fig. 2N; Table 2). The total amount of citric acid in the ejaculate was significantly lower in TP3 when compared with TP1 (36%) (Fig. 2O; Table 2).

## Discussion

Several lifestyle factors have been related to male reproductive problems^5^,^6^,^16^,^17^. However, studies addressing the effects of toxic substances on sperm present controversial results and are often biased^5^,^18^,^19^. Attempting to overcome these problems, the academic festivities were used as a study model, since numerous lifestyle alterations occur at the same time during 7 days (Fig. 1). This model offers exceptional conditions for studying the impact of acute lifestyle changes in a young and healthy population.

**Figure 3:**
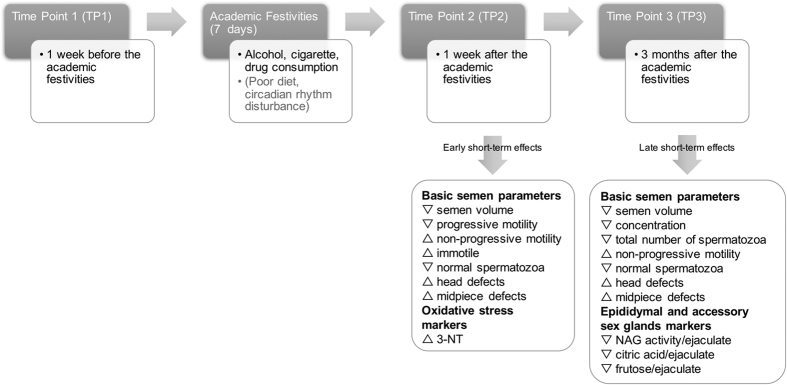
Early (TP2) and late (TP3) short-term impact on seminal quality of the abusive behaviors to which volunteers were exposed during academic festivities week. TP, time-points.

Our study indicates that acute alcohol and cigarette consumption, together with other abusive behaviors to which volunteers exposed themselves during the academic festivities week, produced significant early (TP2) and late (TP3) short-term effects on seminal quality (Figs 2 and 3). As expected, alcohol and cigarette consumption increased during the academic festivities, when compared with values observed one week prior and three months after the academic festivities (Fig. 2). This abrupt lifestyle change was concurrent with deleterious effects on semen quality, both at early and late short-term. Semen volume, spermatozoa concentration, total number of spermatozoa, progressive motility and normal morphology were decreased, along with NAG activity and the amount of fructose and citric acid in the ejaculate. Additionally, the percentage of non-progressive and immotile spermatozoa, together with protein nitration levels were increased (Figs 2 and 3).

It has been reported that chronic alcohol and cigarette consumption can lead to a malfunction of accessory glands^20^,^21^. Our results suggest a late short-term negative impact of the acute lifestyle changes on both epididymal and accessory sex glands function, which is also reflected by a reduction on semen volume throughout the TPs.

Lower levels of seminal vesicles and prostate biomarkers – fructose and citric acid, respectively – were found in TP3 when compared with both TP1 and TP2 (Fig. 2). The seminal vesicles provide the majority of ejaculate volume^22^, thus the impairment of this gland can explain the decrease in volume observed over the TPs (Fig. 2). The prostate provides secretions with zinc, citric acid, prostate specific antigen (PSA), choline and small membrane-bound vesicles called prostasomes^23^,^24^,^25^. The presence of prostasomes has been associated with beneficial effects on sperm motility^26^,^27^. The impairment of the prostate at TP3 can in part explain the negative effects observed in spermatozoa motility (Fig. 2). NAG is secreted by the epididymal epithelium, mainly to provide ideal levels of energy for sperm maturation^28^. The decrease observed on this biomarker at TP3 suggests a late short-term effect of alcohol and nicotine consumption in epididymal function. Previous studies demonstrated that smokers show a marked reduction in NAG^29^, which may be associated with defective sperm maturation, leading to a decrease in sperm motility. Furthermore, lower levels of this enzyme were found in men with oligozoospermia and azoospermia of primary testicular origin^30^. Alcohol consumption was previously associated with reduced epididymis weight^31^, an increased infiltration of intact mast cells and a reduced index of degranulated mast cells in rat epididymis^20^. The fact that motility is acquired in the epididymis and acute abusive alcohol and cigarette consumption compromised epididymis function at TP3 may explain their mid-term harmful effects in spermatozoa motility (Fig. 2).

To evaluate the damage to sperm cells caused by ROS we checked for the presence of 3-NT groups. These molecules are products of the tyrosine nitration, mediated by peroxynitrite (ONOO-) which, in turn, is produced by the reaction of O2•– and NO^32^. An increase of 3-NT was observed in TP2 (Fig. 2), indicating that the acute lifestyle changes were responsible for increasing ROS levels in spermatozoa and, consequently, for an effect on protein modifications by tyrosine nitration. Excessive levels of protein nitration may lead to alterations in protein function and structure, which may result in a compromise of sperm function. In fact, Morielli and O’Flaherty demonstrated that oxidative stress promotes a dose-dependent increase of tyrosine nitration and alters motility and the ability of spermatozoa to undergo capacitation^33^. This may, in part, explain the deleterious effects observed in motility and morphology parameters at TP2 (Fig. 2). Nevertheless, alcohol may be directly affecting spermatozoa motility, specifically progressive motility, straight line and curvilinear velocity^34^. Also, ROS can damage spermatozoa biomolecules contributing to the increase in abnormal forms and induce sperm apoptosis, explaining the reduction in sperm concentration.

The present study also shows that acute lifestyle alterations, mostly by increasing alcohol and cigarette consumption, have a significant effect on spermatozoa morphology. Increased head and midpiece defects and decreased percentage of morphologically normal spermatozoa were observed a week (TP2) and 3 months (TP3) after the acute lifestyle changes, suggesting an effect on already morphologically mature spermatozoa in the epididymis and on spermatozoa forming in the testicles (Fig. 2). A study showed that very few alcoholic men are normozoospermic, which reinforces the deleterious effects of alcohol in spermatozoa morphology^5^. Both spermatozoa concentration and the percentage of morphologically normal spermatozoa were decreased 3 months after the academic festivities, suggesting that the spermatogenesis process was affected.

The power of the study is limited by the relatively small overall sample size and by the risk of misclassification bias due to the use of retrospective information on alcohol, cigarette and drugs exposure. Selection bias has also to be considered, since all volunteers were Caucasians. Furthermore, we cannot exclude that our findings are due to unmeasured confounders, including diet, circadian rhythm alterations, exercise and stress.

Due to the usual behavior of the population attending the academic festivities it was very difficult to recruit subjects not altering their lifestyle in TP2. In fact, in our sample, only 2 individuals fit this criterion. However, one can observe that the overall tendency of the results point to a detrimental effect of the lifestyle alterations on seminal quality. If the results were due to the natural variation in semen analyses, a random pattern of changes would be expected.

The minimum sample size was not calculated due to the lack of information about the population. However, the dataset size was considered reasonable since, according to the central limit theorem, it was possible to approximate the distribution of variables to a normal distribution.

This study contributed to further understanding of the pathophysiology underlying the semen quality degradation induced by acute lifestyle alterations, suggesting that specifically high alcohol and cigarette consumption, are associated with decreased semen quality in healthy young men, being correlated with detrimental effects on all seminal parameters, increase in spermatozoa oxidative stress and compromised function of the epididymis and accessory sex glands. Healthy men may therefore be advised that occasional acute lifestyle alterations may harm their reproductive health at short-term. Further studies are needed to understand whether the seminal quality recovers or if there is an actual long-term impact of acute lifestyle alterations.

## Additional Information

**How to cite this article:** Vieira Silva, J. *et al*. Study on the short-term effects of increased alcohol and cigarette consumption in healthy young men’s seminal quality. *Sci. Rep.* **7**, 45457; doi: 10.1038/srep45457 (2017).

**Publisher's note:** Springer Nature remains neutral with regard to jurisdictional claims in published maps and institutional affiliations.

## Supplementary Material

Supplementary Table S1

## Figures and Tables

**Table 1:** Descriptive analyses of questionaries’ data, basic semen parameters, oxidative stress markers, and epididymal and accessory sex glands function markers at the different time points (TP).

	N	TP1				TP2				TP3			
		Mean	SD	Minimum	Maximum	Mean	SD	Minimum	Maximum	Mean	SD	Minimum	Maximum
Alcohol consumption (g)	36	15.2	32.2	0.0	179.2	123.0	130.5	0.0	480.0	18.4	24.4	0.0	96.0
Nicotine consumption (mg)	36	1.0	2.4	0.0	8.4	2.0	3.8	0.0	14.4	1.3	2.9	0.0	12.0
Age (years)	36	22	3	18	32								
Sexual abstinence (days)	32	2	3	0	10	2	3	0	13	3	4	0	20
Volume (mL)	36	3.9	1.9	1.0	9.0	3.2	2.0	0.7	9.0	2.6	1.4	1.0	6.0
Concentration (x10^6^/mL)	36	61	37	20	155	53	39	7	194	49	41	10	241
Total number spermatozoa (x10^6^)	36	236	189	40	775	169	198	16	1062	114	84	23	362
Progressive motility (%)	36	54	15	21	89	46	21	14	91	48	19	4	72
Non-progressive motility (%)	36	10	5	0	21	14	9	1	37	15	7	4	38
Immotile (%)	36	36	14	9	74	41	18	8	71	37	20	11	81
Normal morphology (%)	36	11	5	3	19	9	4	2	22	9	3	3	18
Head defects (%)	36	65	16	29	95	74	14	37	94	67	12	31	88
Midpiece defects (%)	36	25	14	6	49	34	14	2	69	32	13	16	60
Tail defects (%)	36	22	16	1	59	25	15	0	60	26	13	6	51
Total antioxidant capacity (TAS) (mmol/l)	22	1.44	0.75	0.27	2.63	1.65	0.64	0.37	2.58	1.69	0.69	0.07	2.52
3-Nitrotyrosine (3-NT) (AU)	24	110.94	130.15	0.42	383.33	138.54	152.94	0.39	419.37	99.88	106.52	0.27	273.19
Superoxide dismutase 1 (SOD1) (AU)	23	2.22	3.50	0.36	15.59	1.79	3.23	0.23	15.95	7.33	18.57	0.12	81.42
Glutathione peroxidase 4 (GPx4) (AU)	22	0.64	1.11	0.07	5.26	0.48	0.44	0.04	1.63	0.60	0.66	0.03	2.67
Carbonyl groups (CG) (AU)	12	130.06	85.27	56.27	362.25	139.43	86.09	76.69	392.99	107.92	35.18	68.01	172.59
Citric acid (mg)	12	13.60	6.30	5.87	25.55	12.10	8.86	4.31	36.74	8.65	3.61	4.24	13.65
Frutose (mg)	12	11.30	7.24	2.31	29.01	10.68	7.72	1.47	25.71	8.18	5.98	2.42	19.06
Neutral alfa-glucosidase (mIU)	12	57.07	45.17	21.27	190.42	47.26	40.98	5.97	138.46	30.90	30.42	6.42	122.74

**Table 2:** Paired difference tests between the study time-points (TPs).

	N	Paired groups	p value (2-tailed)	Effect size Cohen’d
Alcohol consumption	36	TP1 vs TP2	0.00^a^	1.1
		TP2 vs TP3	0.00^a^	1.1
Nicotine consumption	36	TP1 vs TP2	0.00^a^	0.3
**Basic semen parameters**				
Volume	36	TP1 vs TP2	0.02^a^	0.4
		TP1 vs TP3	0.00^a^	0.8
		TP2 vs TP3	0.04^a^	0.3
Concentration	36	TP1 vs TP3	0.04^a^	0.3
Total number of spermatozoa	36	TP1 vs TP3	0.00^a^	0.8
Progressive motility	36	TP1 vs TP2	0.01^a^	0.4
Non-progressive motility	36	TP1 vs TP2	0.04^a^	0.5
		TP1 vs TP3	0.002^a^	0.8
Immotile	36	TP1 vs TP2	0.05^a^	0.3
Normal morphology	36	TP1 vs TP2	0.00^a^	0.4
		TP1 vs TP3	0.00^a^	0.5
Head defects	36	TP1 vs TP2	0.00^a^	0.6
		TP2 vs TP3	0.001^a^	0.5
Midpiece defects	36	TP1 vs TP2	0.00^a^	0.6
		TP1 vs TP3	0.00^a^	0.5
**Oxidative stress markers**				
3-Nitrotyrosine (3-NT)	24	TP2 vs TP3	0.01^b^	0.3
**Epididymal and accessory sex glands markers**				
Citric acid	12	TP1 vs TP3	0.03^a^	1.0
Frutose	12	TP1 vs TP3	0.02^a^	0.5
		TP2 vs TP3	0.04^a^	0.4
Neutral α-glucosidase	12	TP1 vs TP3	0.02^a^	0.7
		TP2 vs TP3	0.04^c^	0.5

^a^T-test; ^b^Wilcoxon Signed Ranks test (asymp. sig.); ^c^Sign test (exact sig.) - Binomial distribution used.
