# Supplementary material for: Study on the short-term effects of increased alcohol and cigarette consumption in healthy young men’s seminal quality
Source: Sci Rep. 2017 Apr 3;7:45457. doi: 10.1038/srep45457 (PMC5377370; doi:10.1038/srep45457)
Supplement: Supplementary Table S1 [file srep45457-s1.pdf]

Longitudinal study on the short-term effects of increased alcohol and cigarette consumption in young men's seminal quality

Joana Vieira Silva[1+], Daniel Cruz[1+], Mariana Gomes[1+], Bárbara Regadas Correia[1], Maria João Freitas[1], Luís Sousa[1], Vladimiro Silva[2], Margarida Fardilha[1*]

[1] Laboratory of Signal Transduction, Department of Medical Sciences, Institute of Biomedicine – iBiMED, University of Aveiro, Campus Universitário de Santiago, 3810-193, Aveiro, Portugal

[2] Ferticentro – Center for Fertility Studies, Praceta Prof. Robalo Cordeiro (Idealmed), Circular Externa de Coimbra, 3020-479 Coimbra, Portugal

+ These authors contributed equally to this work

* Corresponding author: Margarida Fardilha, Departamento de Ciências Médicas, Agra do Crasto, Campus de Santiago, Universidade de Aveiro, 3810-193 Aveiro, Portugal. T: +351-918143947. E: mfardilha@ua.pt

Supplementary Table S1. Paired difference tests between the study time-points (TPs).

	N	Paired Samples	p value (2-tailed)	Effect size Cohen'd
Sexual abstinence	32	TP1 vs TP2	0.88[a.]	0.0
		TP1 vs TP3	0.25[a.]	0.3
		TP2 vs TP3	0.29[a.]	0.3
Volume	36	TP1 vs TP2	0.02[a.]	0.4
		TP1 vs TP3	0.00[a.]	0.8
		TP2 vs TP3	0.04[a.]	0.3
Concentration	36	TP1 vs TP2	0.21[a.]	0.2
		TP1 vs TP3	0.04[a.]	0.3
		TP2 vs TP3	0.57[a.]	0.1
Total number of spermatozoa	36	TP1 vs TP2	0.05[a.]	0.3
		TP1 vs TP3	0.00[a.]	0.8
		TP2 vs TP3	0.10[a.]	0.3
Progressive motility	36	TP1 vs TP2	0.01[a.]	0.4
		TP1 vs TP3	0.07[a.]	0.4
		TP2 vs TP3	0.52[a.]	0.1
Non-progressive motility	36	TP1 vs TP2	0.04[a.]	0.5
		TP1 vs TP3	0.002[a.]	0.8
		TP2 vs TP3	0.54[a.]	0.1
Immotile	36	TP1 vs TP2	0.05[a.]	0.3
		TP1 vs TP3	0.77[a.]	0.1
		TP2 vs TP3	0.21[a.]	0.2
Normal morphology	36	TP1 vs TP2	0.00[a.]	0.4
		TP1 vs TP3	0.00[a.]	0.5
		TP2 vs TP3	1.00[a.]	0.0
Head defects	36	TP1 vs TP2	0.00[a.]	0.6
		TP1 vs TP3	0.22[a.]	0.1
		TP2 vs TP3	0.001[a.]	0.5
Midpiece defects	36	TP1 vs TP2	0.00[a.]	0.6
		TP1 vs TP3	0.00[a.]	0.5
		TP2 vs TP3	0.20[a.]	0.1
Tail defects	36	TP1 vs TP2	0.28[a.]	0.2
		TP1 vs TP3	0.08[a.]	0.3
		TP2 vs TP3	0.30[a.]	0.1
Alcohol consumption	36	TP1 vs TP2	0.00[a.]	1.1
		TP1 vs TP3	0.48[a.]	0.1
		TP2 vs TP3	0.00[a.]	1.1
Nicotine consumption	36	TP1 vs TP2	0.00[a.]	0.3
		TP1 vs TP3	0.21[a.]	0.1
		TP2 vs TP3	0.08[a.]	0.2
Total antioxidant capacity (TAS)	22	TP1 vs TP2	0.07[b.]	0.3
		TP1 vs TP3	0.21[a.]	0.3
		TP2 vs TP3	1.00[b.]	0.1
3- Nitrotyrosine (3-NT)	24	TP1 vs TP2	0.24[b.]	0.2
		TP1 vs TP3	0.08[b.]	0.1
		TP2 vs TP3	0.01[b.]	0.3
Superoxide dismutase 1 (SOD1)	23	TP1 vs TP2	0.68[c.]	0.1
		TP1 vs TP3	0.68[c.]	0.4
		TP2 vs TP3	1.00[c.]	0.4
Glutathione peroxidase 4 (GPx4)	20	TP1 vs TP2	0.41[c.]	0.2
		TP1 vs TP3	0.84[c.]	0.0
		TP2 vs TP3	0.84[c.]	0.2
Carbonyl groups (CG)	12	TP1 vs TP2	0.39[a.]	0.1
		TP1 vs TP3	0.41[a.]	0.3

		TP2 vs TP3	1.00[c.]	0.5
Citric acid	12	TP1 vs TP2	0.21[b.]	0.2
		TP1 vs TP3	0.03[a.]	1.0
		TP2 vs TP3	0.77[c.]	0.5
Frutose	12	TP1 vs TP2	0.76[a.]	0.1
		TP1 vs TP3	0.02[a.]	0.5
		TP2 vs TP3	0.04[a.]	0.4
Neutral alfa-glucosidase	12	TP1 vs TP2	0.28[a.]	0.2
		TP1 vs TP3	0.02[a.]	0.7
		TP2 vs TP3	0.04[c.]	0.5

a. T-test; b. Wilcoxon Signed Ranks test (asymp. sig.); c. Sign test (exact sig.) - Binomial distribution used.
